# Editorial: Updates on convalescent plasma and monoclonal antibody therapies for infectious disease in patients with primary immunodeficiency

**DOI:** 10.3389/fimmu.2022.1010072

**Published:** 2022-08-26

**Authors:** Antonio Condino-Neto, Andrew R. Gennery

**Affiliations:** ^1^ Department of Immunology, Institute of Biomedical Sciences, University of São Paulo, São Paulo, Brazil; ^2^ Translational and Clinical Research Institute, Newcastle University, Newcastle upon Tyne, United Kingdom; ^3^ Paediatric Stem Cell Transplant Unit, Great North Children’s Hospital, Newcastle upon Tyne, United Kingdom

**Keywords:** predominantly antibody deficiencies, treatment challenges, antibody defects, cellular defects, combined immunodeficiencies

Inborn errors of immunity (IEI) are rare inherited diseases of the immune system. The most recent International Union of Immunological

Societies (IUIS) classification lists 485 diseases. Many patients are susceptible to infectious diseases, which remain a significant cause of morbidity and mortality. Although antimicrobial agents can be effective at helping clear pathogenic organisms, for many patients with IEI, particularly those who harbor viral infection, defective immunity may mean that they excrete virus for a prolonged period. The SARS-CoV-2 pandemic demonstrated that patients with IEI who contract Covid19 are at risk from significant sequelae and some continue to excrete virus for many weeks, potentially leading to viral mutations and the development of new strains. Naturally occurring antibodies have a role in the clearance of viral infection, but in this patient group, individuals may be unable to initiate, complete or sustain an effective immune response against naturally occurring or vaccine-strain SARS-CoV-2. This Frontiers topic examines the effect of patients with IEI who receive convalescent plasma or monoclonal antibodies therapies.

In a small multi-centre matched-cohort retrospective study from China (Pan et al.), the effects of convalescent plasma, harvested from patients who recovered from COVID-19, on the 60-day mortality and negative conversion rate of SARS-CoV-2 during hospitalization of patients with severe, life-threatening COVID-19 infection was assessed. Patients who received convalescent plasma therapy were matched by age, sex, diabetes, hypertension, heart failure, onset of symptoms to hospital admission, respiratory support pattern, lymphocyte count, troponin, Sequential Organ Failure Assessment (SOFA), glucocorticoid, and antiviral agents to up to three patients with COVID-19 who did not receive convalescent plasma therapy. Cox regression and competing risk analysis were used to assess the effects of convalescent plasma therapy on these patients. Twenty-six patients in the convalescent plasma therapy group were compared with 78 control patients, whose demographic characteristics were similar except for the SOFA score, which was higher in the former group. Treatment with convalescent plasma therapy did not improve 60-day mortality but the SARS-CoV-2 negative conversion rate for 60 days after admission was higher in the treatment group. These patients did not have an IEI.

The second, larger, longitudinal multi-centre study, from Italy (Garzi et al.), specifically looked at outcomes of 192 patients with an IEI, previously vaccinated with mRNA vaccines, infected with SARS-CoV-2, and treated either as an inpatient, or an outpatient. The impact of monoclonal antibody administration and antiviral treatment on the risk of mortality, hospital admission, and severe disease was evaluated, as well as the safety of monoclonal antibodies and antiviral drugs in patients with IEI. This was a real life observational study from February 2020-April 2022, and patients were not randomized to receive either treatment or no treatment at all. Different treatments were administered according to availability at the time of infection. Non-treated patients either refused treatment or treatments were unavailable at time of presentation. From March 2021 - December 2021, bamlanivimab or the combination monoclonal antibodies casirivimab/imdevimab and bamlanivimab/etesivimab were administered. From December 2021, Sotrovimab was given. From March-October 2020 lopinavir/ritonavir and darunavir/ritonavir were administered. From October 2020, remdesivir was given to hospitalized patients and from November 2021 molnupiravir and ritonavir were available. Analyses of efficacy were performed according to the different circulating SARS-CoV-2 strains. The original Wuhan strain was isolated February-December 2020, the B.1.1.7 (Alpha) variant January 2021 - mid-July 2021, the B.1.617.2 (Delta) variant from mid-July 2021 - December 2021, the B.1.1.529 (BA.1), (Omicron) variant became largely predominant from December 2021 being replaced by BA.2 in March 2022. The study demonstrated that infected patients treated with monoclonal antibodies and/or antivirals had a lower risk of COVID-19-related hospitalization, development of severe disease and death. When analyzed separately, monoclonal antibodies had a advantageous affect on risk of hospitalization and severe disease. Antiviral use showed a slight impact only on the risk of hospitalization only. Adjusting for age and sex in the whole cohort of patients infected during the entire study period confirmed that treatment with monoclonal antibodies and/or antivirals had reduced the risk of hospitalization but had no significant effect on the occurrence of severe disease and death. No significant adverse events were recorded. The authors conclude that administration of monoclonal antibodies and/or antivirals has a beneficial effect in this at risk population, but that the rapid spread of new viral strains underlines that beneficial effects of treatment should be re-evaluated over time.

Case reports are less accurate at answering a hypothesis, but can give valuable insights. One reports an adult patient, previously undergoing haematopoietic stem cell transplantation (HSCT) for severe combined immunodeficiency as an infant, and having poor graft function, awaiting further HSCT, who contracted SARS-CoV-2 (Keitel et al.). The viral load decreased initially after administration of remdesevir, but rose again, and cleared after receiving 6 doses of convalescent plasma from 2 different donors. The patient subsequently had a successful HSCT without recurrence of SARS-CoV-2.

A second report describes an adult with activated PI3K-kinase delta syndrome, who was treated for a diffuse large B cell lymphoma with R-CHOP, and acquired SARS-CoV-2 within 1 month of completing therapy (Rivalta et al.). Initially asymptomatic, he continued to be viral PCR positive, and upon developing symptoms of low-grade fever, with dry cough, and exertional dyspnea and worsening of pulmonary consolidation on imaging, he received 10 days treatment with remdesivir. He remained SARS-CoV-2 positive by PCR. He reacted to an infusion of convalescent plasma, which was discontinued, but tolerated casirivimab and imdevimab SARS-CoV-2-neutralizing monoclonal antibodies, and was subsequently SARS-CoV-2 PCR negative.

These studies and reports suggest that convalescent sera and monoclonal antibodies therapies have a role in treating patients with IEI who develop infection with SARS-CoV-2. Given the changing nature of the pandemic, with viral mutation, studies will need to continue as different strains evolve and new monoclonal antibodies are developed, for example Tixagevimabe and Cilgavimabe given together prophylactically for pre-exposure or early stage post exposure to SARS-COV2 virus, for IEI or other patients with secondary immunodeficiencies due to cancer, transplants, leukemia or lymphomas (1).

## Author contributions

AC-N and AG reviewed the published papers on the Research Topic and wrote the manuscript. Both authors critically evaluated, edited and approved the submitted article.

## Conflict of interest

The authors declare that the research was conducted in the absence of any commercial or financial relationships that could be construed as a potential conflict of interest.

## Publisher’s note

All claims expressed in this article are solely those of the authors and do not necessarily represent those of their affiliated organizations, or those of the publisher, the editors and the reviewers. Any product that may be evaluated in this article, or claim that may be made by its manufacturer, is not guaranteed or endorsed by the publisher.
